# Evaluation of a novel tomographic ultrasound device for abdominal examinations

**DOI:** 10.1371/journal.pone.0218754

**Published:** 2019-06-26

**Authors:** Valentin Blank, Johannes Wiegand, Volker Keim, Thomas Karlas

**Affiliations:** 1 Division of Gastroenterology, University Hospital Leipzig, Leipzig, Germany; 2 Integrated Research and Treatment Center Adiposity Diseases, University of Leipzig, Leipzig, Germany; Medizinische Fakultat der RWTH Aachen, GERMANY

## Abstract

Conventional ultrasound (US) is the first-line imaging method for abdominal pathologies, but its diagnostic accuracy is operator-dependent, and data storage is usually limited to two-dimensional images. A novel tomographic US system (Curefab CS, Munich, Germany) processes imaging data combined with three-dimensional spatial information using a magnetic field tracking. This enables standardized image presentation in axial planes and a review of the entire examination. The applicability and diagnostic performance of this tomographic US approach was analyzed in an abdominal setting using conventional US as reference. Tomographic US data were successfully compiled in all subjects of a training cohort (20 healthy volunteers) and in 50 patients with abdominal lesions. Image quality (35% and 79% for training and patient cohort respectively) and completeness of organ visualization (45% and 44%) were frequently impaired in tomographic US compared to conventional US. Conventional and tomographic US showed good agreement for measurement of organ sizes in the training cohort (right liver lobe and both kidneys with a median deviation of 5%). In the patient cohort, tomographic US identified 57 of 74 hepatic or renal lesions detected by conventional ultrasound (sensitivity 77%). In conclusion, this study illustrates the diagnostic potential of abdominal tomographic US, but current significant limitations of the tomographic ultrasound device demand further technical improvements before this and comparable approaches can be implemented in clinical practice.

## Introduction

Ultrasound is the first line method for detection and characterization of abdominal pathologies, especially focal lesions in screening and follow-up scenarios [1]. However, the diagnostic value of conventional ultrasound is highly operator-dependent. Moreover, the documentation of ultrasound examinations is traditionally performed in poorly standardized two-dimensional images [2–4]. This neither permits a retrospective review of the entire examination nor allows a sufficient comparison with radiological imaging (computed tomography, CT; magnetic resonance imaging, MRI).

These limitations of conventional ultrasound can be overcome by recording and processing of three-dimensional (3D) ultrasound data, which allow presentation, analysis and storage in standardized planes. Different approaches of 3D ultrasound have been developed and extensively studied in the past decade [5]. Dedicated 3D/4D US probes (mechanical 3D probes) include a rotational tilt transducer unit that scans an area of interest within an angle up to 90° and allows complex processing of the image volume data [5, 6]. These probes are widely applied in gynaecology and obstetrics [7] as well as in echocardiography [8], and have also been evaluated for differential diagnosis of liver lesions [9, 10]. Although these studies report a high image quality especially for vascular architecture of liver lesions, the use of 3D/4D US probes for abdominal imaging is limited to predefined areas of restricted size. Besides 3D/4D volume probes, different types of linear mechanical scanning mechanisms have been suggested [6], but have not yet been implemented in clinical use for abdominal imaging.

Recently, a new tomographic ultrasound system has been developed that combines spatial tracking of the ultrasound probe position with image data from high-class ultrasound devices [11, 12]. This technology enables the free-hand recording of large metrical 3D-volumes covering whole organs and offers the possibility of ultrasound data post-processing in analogy to CT and MRI. Pilot studies showed a promising performance for diagnosis and characterization of complex vascular stenosis [13–15] and for detection of thyroid nodules [16].

In the present pilot study, we evaluated the applicability and diagnostic performance of the tomographic ultrasound for abdominal examinations. We found a good metrical concordance with measurements of conventional ultrasound, but limitations in image quality and diagnostic accuracy for detection of focal lesions.

## Patients and methods

### Study aim

This pilot study was designed to compare

1. the applicability, image quality and metric comparability of tomographic ultrasound, and
2. the diagnostic accuracy of the tomographic ultrasound for the detection of focal lesions in abdominal organs (liver and kidney)

with conventional ultrasound as reference standard.

### Study population

**Fig 1:**
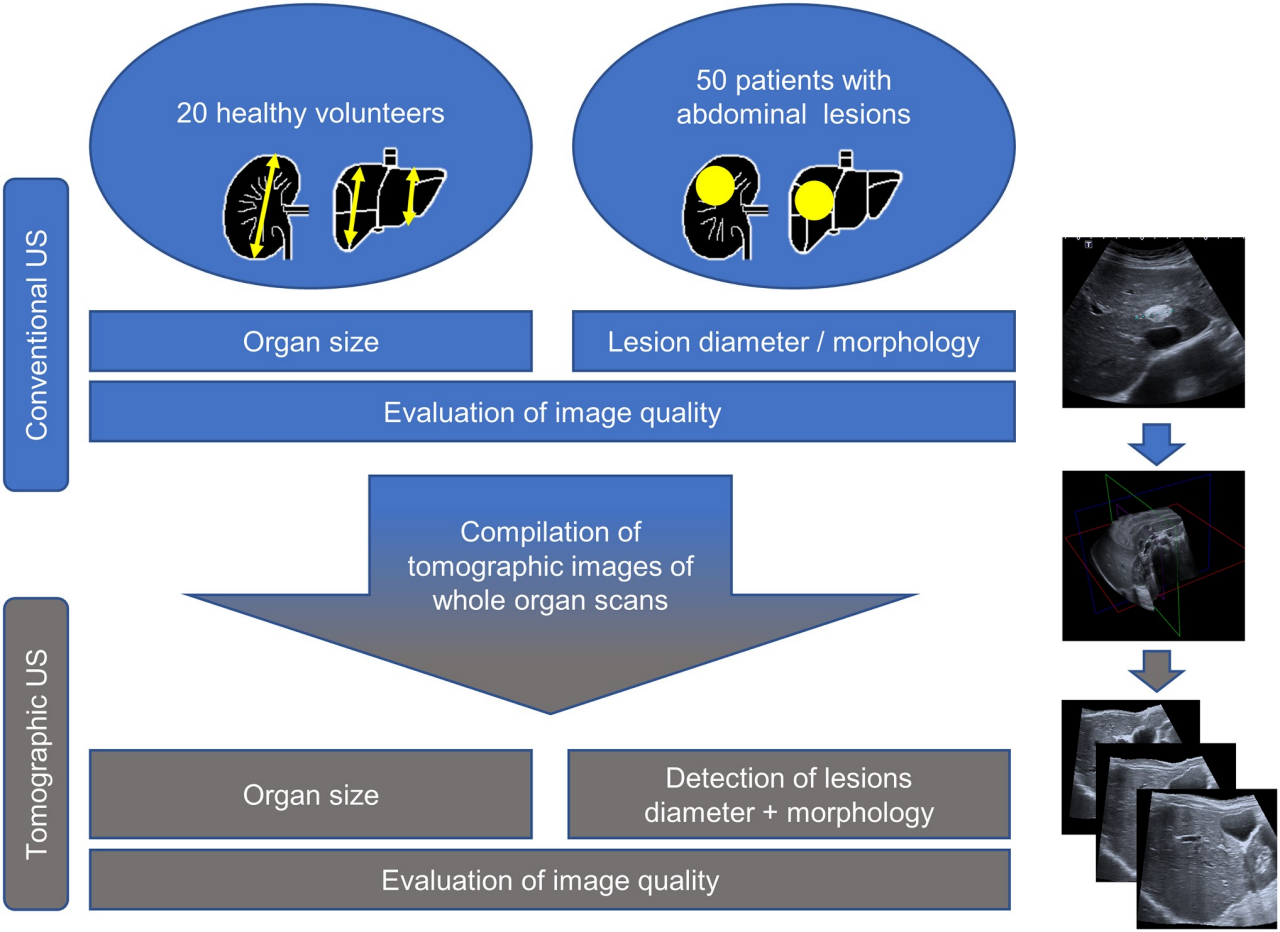
Overview of study concept.

Fig 1 gives an overview of the study protocol. For studying the applicability and technical precision, we performed tomographic US in healthy volunteers with normal body weight and without any history of chronic diseases (training cohort).

For assessment of the diagnostic performance, consecutive patients (patient cohort) presenting to our ultrasound diagnostic unit for abdominal examination were prospectively recruited for the study, if the following criteria were fulfilled: patients were eligible if at least one focal cystic or tumorous lesion of the liver or kidney with a size > 5 mm could be clearly visualized by a routine clinical ultrasound performed by experienced DEGUM certified examiners (VK, TK). Exclusion criteria comprised age <18 years, pregnancy, and failure to apply a complete standard conventional ultrasound (e.g. in cases with abdomen apertum or drainages).

The study was approved by the local institutional review board (Ethical Committee at the Medical Faculty, Leipzig University, reg. no. 352-08-B-ff). All participants provided written informed consent. The patients and healthy volunteers were recruited for the study from March to June 2016.

### Ultrasound diagnostics

**Fig 2:**
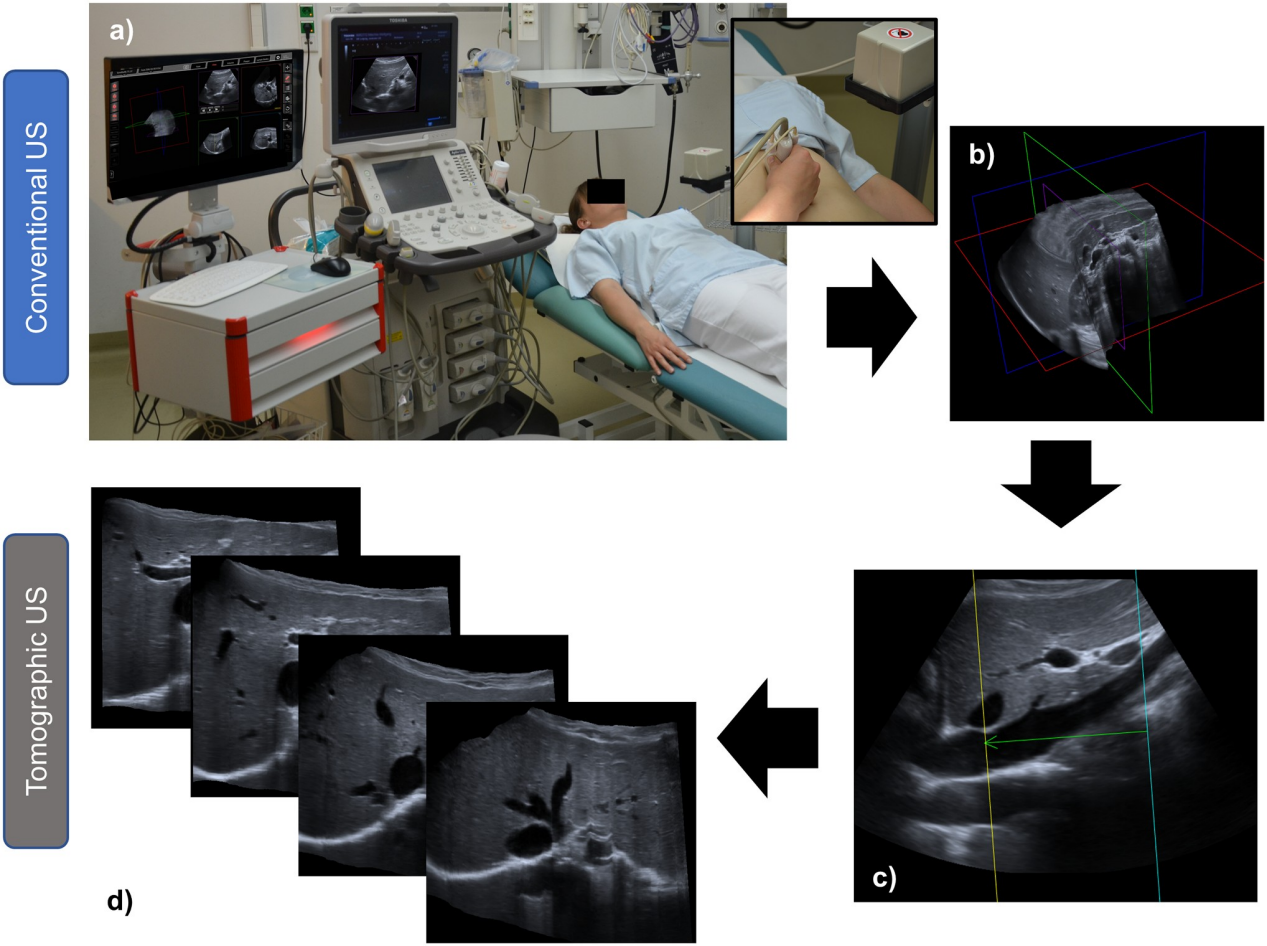
Acquisition of tomographic ultrasound. Conventional ultrasound image-data and spatial information from the tracking system (a) were continuously recorded during a whole organ scan. The tomographic volume (b) was alined in sagittal orientation (c) and series of transversal planes were computed (d). The person (a) gave written permission to use this photo.

1. **Conventional ultrasound**
  All participants underwent a structured conventional abdominal ultrasound examination using a state-of-the-art ultrasound device (Aplio 500, Toshiba Medical Systems, Germany) equipped with a curved-array transducer (6C1 PVT-375 BT 3–6 MHz). In a first step, the reference examiners (VK, TK) used standard ultrasound planes and images to document
  - organ morphology and size in the training cohort: Both liver lobes were measured separately (left liver lobe: anterior-posterior diameter in the median line; right liver lobe: anterior-posterior diameter in the medio-clavicular-line). For both kidneys, the maximal diameter was recorded.
  - the number, morphology and largest diameter of all detected lesion of liver and/or kidney(s) in the patient cohort.
  In addition to these metric measurements, image quality and completeness of organ recording were assessed in both cohorts using ordinal scales.
2. **Tomographic ultrasound**
  Immediately after the conventional ultrasound examination, three-dimensional (3D) ultrasound scans of right and left liver lobe and kidneys were recorded by the same examiner using a computed tomographic sonography system (Curefab Technologies GmbH, Munich, Germany). This device comprised a freehand field tracking system with spatial sensors attached to the probe, a magnetic field generator, and a workstation equipped with a software package for generating 3D-volumes (Curefab CS, version 1.10.0; S1 Video). In brief, two-dimensional image information was continuously gripped from the video port (DVI) of the ultrasound device and compiled with the spatial information of the magnetic field sensors [12]. The examiner aimed to perform one large scan covering the whole kidney or liver lobe. The attached computer system then generated a 3D-volume, which could be orientated according to the patient sagittal axis and, hence, compile multiple transversal image planes (Fig 2; S2 Video). The total tomographic US scan including image processing took approximately five minutes per organ.

### Blinded review of tomographic ultrasound

Subsequently, the 3D volumes and transversal image planes were reviewed by a different examiner (VB, DEGUM certified examiner with special training with the 3D application) who was blinded to all clinical and conventional ultrasound information. This examiner now used the tomographic data to

1. measure organ sizes (training cohort), and
2. to detect liver and/or kidney lesions in the patient cohort. Notably, he was not aware of neither the number nor the locations of these lesions in the conventional US. For all detected lesions ≥ 5 mm, diameter and morphology were characterized.

In addition, image quality and completeness of organ recording in the tomographic scans were documented as described above.

### Analysis of image data and observer-agreement

Agreement in ultrasound scan quality between conventional and tomographic US was compared using ordinal scales:

- image quality: “good = as to be expected from a high-end US device; sufficient = considerable artefacts, but still appropriate for diagnosis; or inappropriate = insufficient quality for diagnosis”.
- completeness of organ recording: “complete”, “margins incomplete”, or “insufficient”.

The rating deviations between both US approaches were analyzed. The evaluation of the conventional image quality was performed by the reference examiner immediately after the conventional US examination. The image quality for the tomographic US scans was assessed by the blinded examiner in the same way. All examiners were trained on the categorial scales prior to the study.

Similarly, the number of detected lesions and their morphology characteristics (“low echogenicity, iso-echogenicity, increased echogenicity, or complex morphology”) were compared between conventional and tomographic US. In few patients, organ lesions were firstly described by the blinded review of tomographic images, i.e. these lesions had not been described by the reference examiner using conventional ultrasound. In such cases, the conventional US video data were again retrospectively reviewed by the original reference examiner. For metric measurements (organ and lesion size), the absolute and relative differences in organ size were calculated and compared.

Sensitivity and specificity of tomographic ultrasound were calculated using conventional ultrasound as diagnostic reference. Categorial data were compared using the Fisher-Yates-test. For non-parametric comparison of independent, not normally distributed data, the Mann-Whitney-U-test was applied. In case of normal distribution, the t-test was used. Statistical analyses were performed with Prism (GraphPad, Version 7). P-values <0.05 indicated as significant difference.

## Results

For the training cohort, 20 healthy volunteers (55% female, median age 28 years, body-mass-index (BMI) 22.2 kg/m^2^) were included. The patient cohort consisted of 50 subjects with liver and/or kidney lesions (60% female, median age 51 years, BMI 26.3 kg/m^2^).

### Applicability and metrical comparability of tomographic US in the training cohort

Tomographic US could be applied successfully in all healthy volunteers. Image quality and completeness of organ scans were rated inferior to conventional US in 35% and 45% of cases, respectively. Notably, image quality of the liver was significantly reduced (right lobe p = 0.001; left lobe p = 0.039), whereas no significant differences were observed for both kidneys (p>0.30, respectively) (Table 1).

**Table 1:** Comparison of tomographic and conventional ultrasound in the training cohort.

	Right liver lobe	Left liver lobe	Right kidney	Left kidney
**Image quality**				
*Conventional US*				
Good	17 (85%)	18 (90%)	17 (85%)	6 (30%)
Sufficient	3 (15%)	2 (10%)	3 (15%)	14 (70%)
Inappropriate	0 (0%)	0 (0%)	0 (0%)	0 (0%)
*Tomographic US*				
Good	8 (40%)	11 (55%)	15 (75%)	4 (20%)
Sufficient	9 (45%)	7 (35%)	4 (20%)	14 (70%)
Inappropriate	3 (15%)	2 (10%)	1 (5%)	2 (10%)
*Tomographic US inferior to the conventional scans*	10 (50%)	7 (35%)	5 (25%)	6 (30%)
**Organ completeness**				
*Conventional US*				
Complete	1 (5%)	5 (25%)	6 (30%)	1 (5%)
Margins incomplete	13 (65%)	14 (70%)	12 (60%)	15 (75%)
Insufficient	6 (30%)	1 (5%)	2 (10%)	4 (20%)
*Tomographic US*				
Complete	0 (0%)	4 (20%)	7 (35%)	2 (10%)
Margins incomplete	3 (15%)	10 (50%)	5 (25%)	6 (30%)
Insufficient	17 (85%)	6 (30%)	8 (40%)	12 (60%)
*Tomographic US inferior to the conventional scans*	11 (55%)	7 (35%)	9 (45%)	9 (45%)
**Measurement agreement of maximal organ diameter**				
Conventional US [mm]*	109 (105–121)^1^	61 (54–67)^2^	112 (109–116)^3^	113 (108–119)^3^
Tomographic US [mm]*	112 (105–124)^1^	70 (63–75)^2^	109 (102–113)^3^	105 (102–112)^3^
Median deviation [%]*	5 (3–9)	13 (5–27)	4 (2–6)	5 (4–10)
Deviation >20%	1 (5%)	6 (30%)	0 (0%)	0 (0%)

*Median(IQR).

^1^ventral-dorsal right kidney.

^2^ventral-dorsal abdominal aorta.

^3^length.

Tomographic and conventional US showed a good accordance in metric assessment of the right liver lobe and both kidneys with a median deviation of 5%. However, a higher deviation was observed for the left liver lobe with a relevant discordance in 30% of cases (Table 1).

### Applicability and diagnostic value of tomographic ultrasound for detection of focal abdominal lesions

Tomographic ultrasound was feasible in all recruited patients, but some cases required repetitive scanning attempts, because the magnetic field tracking interfered with metal objects such as the stretcher or infusion pumps. The image quality of tomographic scans was significantly inferior to the conventional examination (p<0.001 for all organs). In addition, the completeness of the scans was significantly impaired in the liver (right lobe: p = 0.006; left lobe: p<0,001) (Table 2).

**Table 2:** Comparison of tomographic and conventional ultrasound in the patient cohort.

	Right liver lobe	Left liver lobe	Right kidney	Left kidney
**Image quality**				
*Conventional US*				
Good	31 (62%)	40 (80%)	42 (84%)	30 (60%)
Sufficient	14 (28%)	10 (20%)	7 (14%)	16 (32%)
Inappropriate	5 (10%)	0 (0%)	1 (2%)	4 (8%)
*Tomographic US*				
Good	3 (6%)	1 (2%)	13 (26%)	4 (8%)
Sufficient	16 (32%)	11 (22%)	20 (40%)	28 (56%)
Inappropriate	31 (62%)	38 (76%)	17 (34%)	18 (36%)
*Tomographic US inferior to the conventional scans*	38 (76%)	47 (94%)	34 (68%)	38 (76%)
**Completeness**				
*Conventional US*				
Complete	3 (6%)	17 (34%)	24 (48%)	15 (30%)
Margins incomplete	12 (24%)	28 (56%)	21 (42%)	17 (34%)
Insufficient	35 (70%)	5 (10%)	5 (10%)	18 (36%)
*Tomographic US*				
Complete	0 (0%)	5 (10%)	21 (42%)	11 (22%)
Margins incomplete	3 (6%)	13 (26%)	16 (32%)	15 (30%)
Insufficient	47 (94%)	32 (64%)	13 (26%)	24 (48%)
*Tomographic US inferior to the conventional scans*	15 (30%)	34 (68%)	17 (34%)	22 (44%)
**Characterization of lesions**				
*Conventional US*				
Number^1^	31	21	8	14
Diameter [mm] *	24(15–34)	23(12–28)	14(12–15)	15(9–20)
*Tomographic US*				
Number^1^	27	23	13	19
Diameter [mm]*	22(17–36)	19(11–30)	13(10–16)	13(8–18)
**Diagnostic performance of tomographic ultrasound**				
Percentage of detected lesions	21/31 (68%)	18/21 (86%)	6/8 (75%)	12/14 (86%)
Diameter detected lesions [mm]*	24 (18–36)	23 (14–29)	12 (11–14)	18 (9–21)
Diameter of missed lesions [mm]*	19 (13–29)	11 (10–12)	15 (15–16)	11 (11–12)
Morphology agreement	11 (52%)	10 (55%)	5 (83%)	9 (75%)

*Median(IQR).

^1^longest lesion of the respective organ.

In the conventional US examination of the patient cohort, a total of 101 lesions were detected by the reference examiners. The largest lesion per each organ/lobe (reference lesion) was further characterized (52 liver lesions and 22 kidney lesions). The blinded review of the tomographic US examination only detected 57 of these lesions (39 liver lesions and 18 kidney lesions) (sensitivity 77%). The mean diameters of the missed pathologies did not differ from correctly identified lesion (20±4 vs. 27±2 mm, p = 0.16, and 13±3 vs. 16±2 mm, p = 0.60, respectively). Patients with missed lesions did not show differences in gender (p = 0.38), BMI (p = 0.67), image quality (p = 0.64) and completeness of the scans (p = 0.32) compared to cases with correctly identified lesions. Morphology agreement was only observed for half of the liver and three-fourths of the kidney lesions (Table 2).

In addition, 22 lesions (diameter 18±3 mm) were de novo detected in the tomographic US data. The review of the conventional US video information by the reference examiner confirmed seven of these lesions (n = 2 liver, n = 7 kidney), whereas 15 putative lesions (n = 6 liver, n = 9 kidney) could not be verified (specificity of tomographic US 89%).

## Discussion

**Fig 3:**
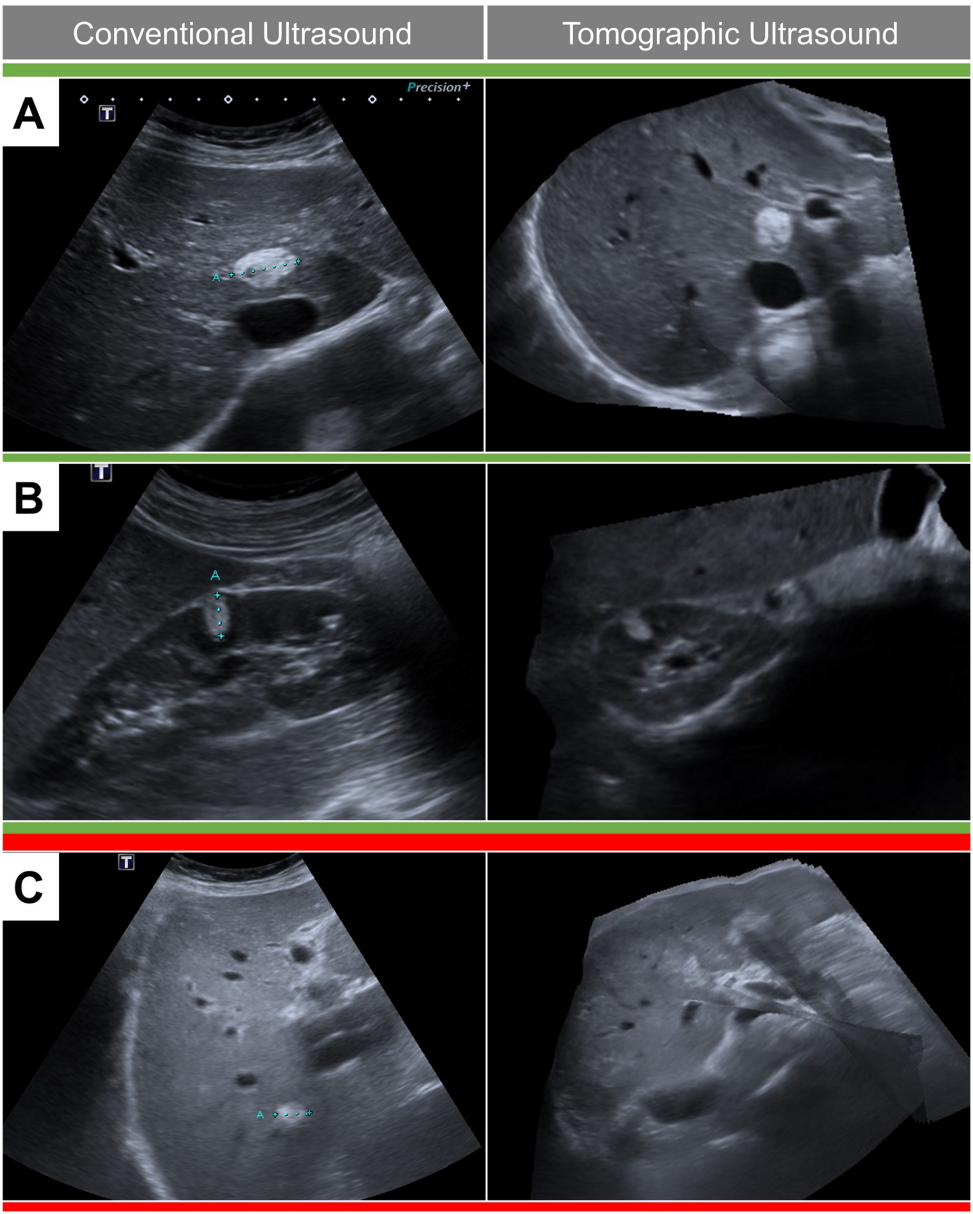
Impact of image quality and artifacts on tomographic ultrasound. Liver and kidney lesion are shown in conventional (left) and tomographic (right) ultrasound. Panels A and B represent examples with good image quality of the tomographic reconstruction. Panel C shows artifacts in the reconstructed image (right) which impairs the detection of the small hyperechoic lesion.

This is the first report on the diagnostic value of a magnetic field tomographic US for the evaluation of abdominal pathologies. Our pilot data demonstrate the diagnostic potential of tomographic US, but also indicate relevant limitations of the current technology. The tomographic US approach already shows satisfying results under optimal conditions, e.g. for detection of focal organ lesions in selected patients with outstanding conventional US image quality (Fig 3A + 3B). This corresponds with recent reports on the application of tomographic US for visualization of homogenous tissue and sharp contrasts, i.e. vascular pathologies [13, 14, 17]. However, the image quality and diagnostic precision in the total patient cohort was rather moderate compared to conventional US. Hence, substantial technical improvements are necessary before tomographic US applications reach the quality standard of conventional US, which is the basic requirement for implementation in regular clinical care.

Metric organ assessment with tomographic US was satisfactory for kidneys but showed relevant deviations from conventional US for the left liver lobe which reflects well-known challenges of standardized liver size assessment. However, abdominal organ assessment with tomographic US was specifically associated with significant limitations in image quality and incomplete visualization of organs (i.e. right liver lobe due to difficult subcostal access), which impaired diagnostic performance for the detection of focal lesions (see example Fig 3C). Of note, only 57 of 74 lesions (sensitivity 77%) were correctly identified, whereas 15 putative lesions (specificity 89%) were observed in the tomographic data sets but could not be verified by the reference examiner.

Based on these findings and the practical experiences with the abdominal application of this tomographic US system, we identified major points for further technical improvement: i) the magnetic field tracking interferes with metallic structures (e.g. the stretcher). Thus, optical tracking systems or gyroscope technologies may provide better alternatives. In addition, tracking systems must compensate motional artifacts caused by free-hand image acquisition. ii) In this study, 3D-reconstructions were based on converted video signal information exported to an external workstation, which were limited to a maximal resolution of 451 x 451 pixels. Analysis of high-resolution ultrasound raw data may improve image resolution and quality but was not available for the Curefab system. The integration of the tomographic application in high-end ultrasound devices may overcome this drawback. Furthermore, the applications of dedicated high resolution US probes with higher frequencies (e.g. for pediatric purpose) could improve the image quality in lean subjects. iii) The acquisition of high-quality ultrasound data is regularly impaired by meteorism and small acoustic windows. Thus, the visualization of larger abdominal compartments would necessitate fusion of multiple volumes generated by different probe positions [18], which is, however, not yet available for abdominal US imaging. In this context, the assembling of the image information must avoid folding and overlap artifacts [18–20]. iv) Image recording and processing with the applied tomographic US system as well as the diagnostic assessment of the tomographic data require a complex and time-consuming workflow. Clinical implication would require faster and easier operating solutions.

**Fig 4:**
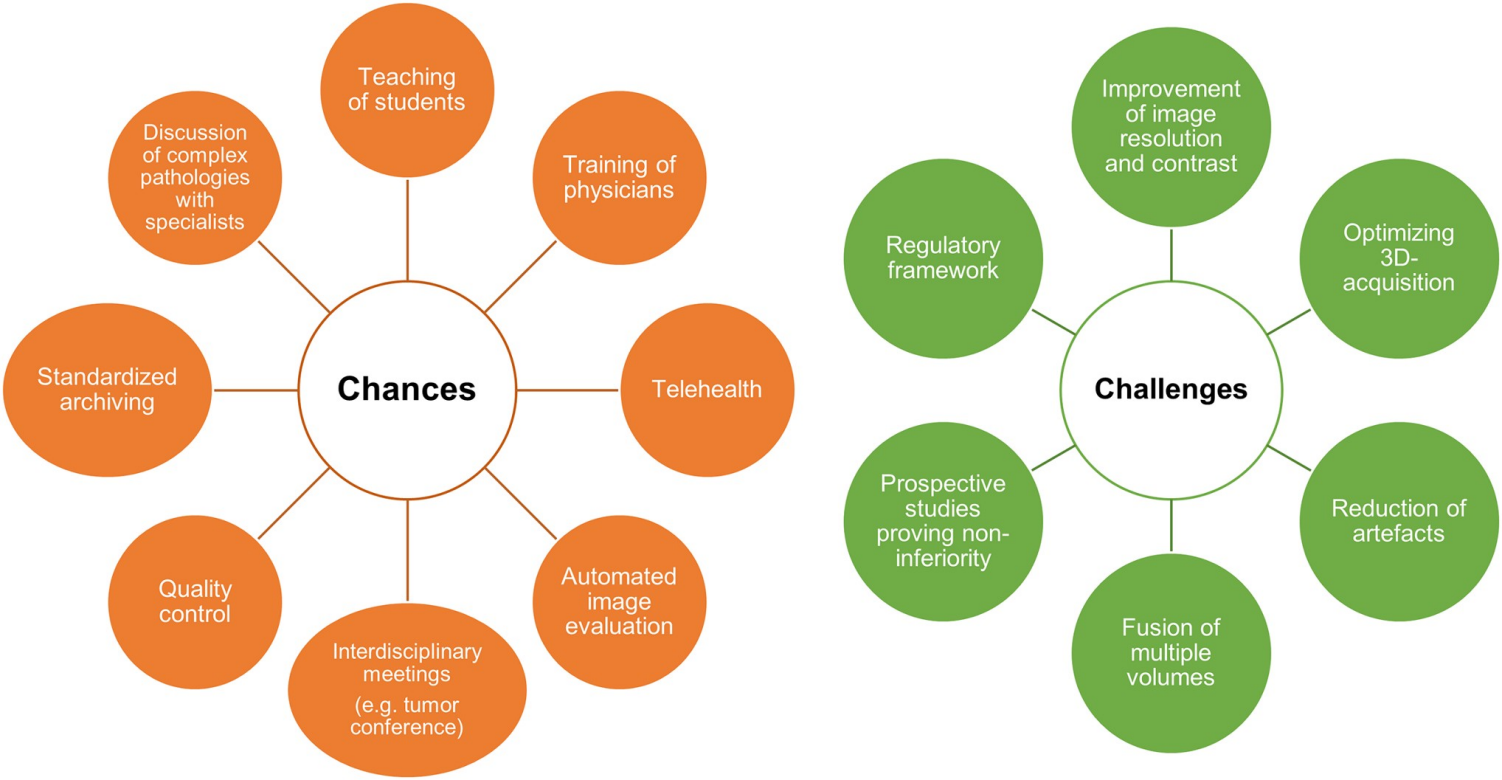
Chances and challenges of three-dimensional ultrasound systems.

The limitations observed in this study represent the specific conditions of the Curefab device. The performance of tomographic ultrasound technologies of other vendors may differ in terms of image resolution and quality but have–to our best knowledge–not yet been systematically studied for abdominal imaging. However, since all commercially available tomographic US systems operate with a similar magnetic tracking system, comparable challenges have to be expected. The outlined limitations and technical issues represent major challenges for developers of ultrasound devices, which can potentially be solved considering the ongoing improvement of hard- and software solutions for 3D image processing [12, 18, 20–23]. The potential clinical and educational applications of tomographic US, e.g. for medical teaching and telehealth concepts [24–26] merit further endeavors in this field (Fig 4).

In addition to these manufacturer specific aspects, our pilot study has some further limitations: First, the diagnostic value of the 3D-image-data relied on the quality of the conventional ultrasound scan which is clearly operator-depended. Due to the pilot character of this study, CT- or MRI-reference-imaging were not available. However, abdominal assessment with the tomographic US approach was clearly rated inferior to conventional scans although all examinations were performed by experienced and certified ultrasound experts. This underlines the need of further improvements of the tomographic US application prior to further diagnostic comparison with advanced radiological imaging and / or invasive diagnostics such as histology. Second, the review of the tomographic US data identified a small number of lesions which were initially not described in the conventional US examination but could eventually be verified. Discordance in number of detected lesions was particularly observed for kidney examination. This organ provides well known diagnostic challenges for ultrasound imaging even when performed by expert examiners [27].

In conclusion, our data highlight the diagnostic potential of tomographic ultrasound for abdominal examination, which may be implemented in various fields of medical education and healthcare. The moderate image quality and the associated limitations of diagnostic accuracy demand further technical improvements before this and comparable approaches can be implemented in clinical practice.

## Supporting information

S1 Video Screenshots of the workstation. Screenshots of the workstation equipped with the 3D-software-package (Curefab CS).
(MP4)

S2 Video Axial slices with focal liver lesions. Sequence of screenshots from axial slices with liver cysts generated by a 3D-volume.
(MP4)
